# A Case of Chronic Eosinophilic Leukemia in a Patient With Recurrent Cough, Dyspnea, and Eosinophilia

**DOI:** 10.7759/cureus.12654

**Published:** 2021-01-12

**Authors:** Nino Balanchivadze, James P Purtell, Jessica Anderson, Yue Guo, Irina Dobrosotskaya

**Affiliations:** 1 Hematology and Oncology, Henry Ford Health System, Detroit, USA; 2 Internal Medicine, Henry Ford Health System, Detroit, USA; 3 Hematology and Oncology, Wayne State University, Detroit, USA; 4 Hematology and Oncology, University of Michigan, Ann Arbor, USA

**Keywords:** hypereosinophilia, chronic eosinophilic leukemia

## Abstract

We report the case of a 40-year-old man with no significant past medical history who had been hospitalized multiple times over the course of one year with recurring cough, dyspnea, pruritic rash, and variable degrees of eosinophilia. He was variably diagnosed with asthma and pneumonia. After his last hospitalization with severe symptoms, the patient was referred for pulmonary evaluation where hypereosinophilia (HE) led to a hematologic workup. Fluorescence in situ hybridization revealed the *FIP1L1-PDGFRA* gene fusion and bone marrow analysis confirmed a diagnosis of chronic eosinophilic leukemia. The patient was treated with daily imatinib and prednisone and he was symptom-free at a four-week follow-up examination.

## Introduction

Peripheral blood eosinophilia, defined as an absolute eosinophil count (AEC) of > 0.5 to 1.0 × 10^9^ cells/L, is common and can occur in various conditions such as infections, allergies, primary hematologic disorders, and other rare entities [1,2]. Hypereosinophilia (HE) is characterized by an AEC of ≥1.5 × 10^9^ cells/L and is associated with more serious conditions. Asthma and atopy can sometimes present as HE, although an AEC ≥5.0 × 10^9 ^cells/L in these cases would be rare [2]. Rapid diagnosis and initiation of therapy for clonal HE are particularly crucial because disease may inevitably progress to an aggressive form [3] and could cause end-organ dysfunction. Importantly, some forms of clonal HE are highly treatable, and therapies can lead to a swift improvement in quality of life; therefore, it is critical that physicians consider hematologic malignancies in the differential diagnosis within the context of recurrent eosinophilia and certain common conditions, when the more obvious causes do not surface.

We report the case of a man with HE who was hospitalized numerous times before being diagnosed with chronic eosinophilic leukemia and whose symptoms significantly improved upon initiation of therapy.

## Case presentation

A 40-year-old man had recurrent hospitalizations with cough, pleuritic chest pain, and a relapsing and remitting pruritic papular rash associated with variable levels of eosinophilia. His symptoms were variously attributed to asthma and pneumonia, although he had no history of asthma. He was hospitalized more than six times over one year and was repeatedly treated with steroids and antibiotics. Additionally, he had undergone multiple non-invasive and invasive testing, including several computed tomograms and cardiac imaging.

**Figure 1:**
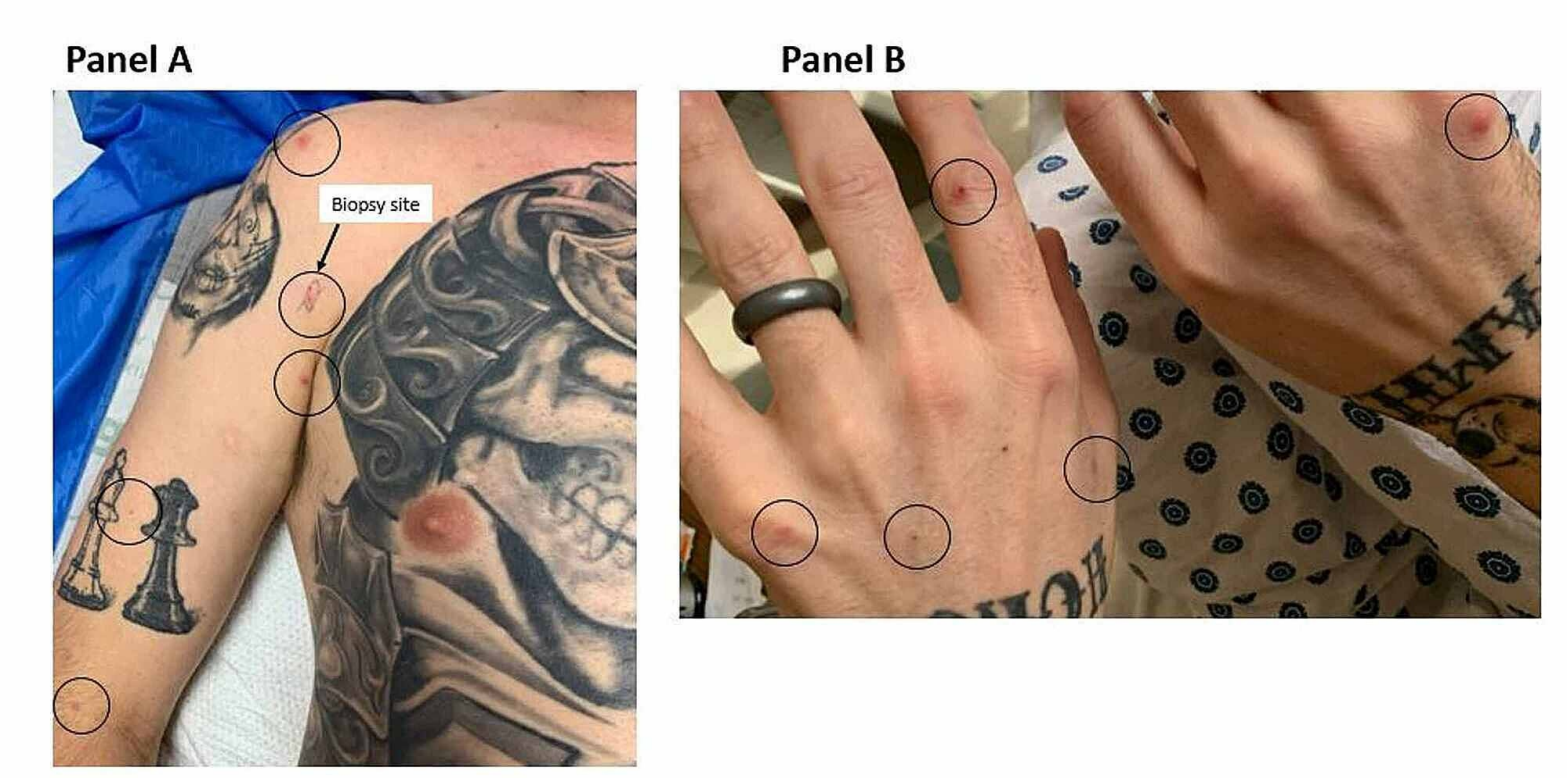
Scattered erythematous papules on his extremities in various stages, some with central ulceration and crusting.

**Figure 2:**
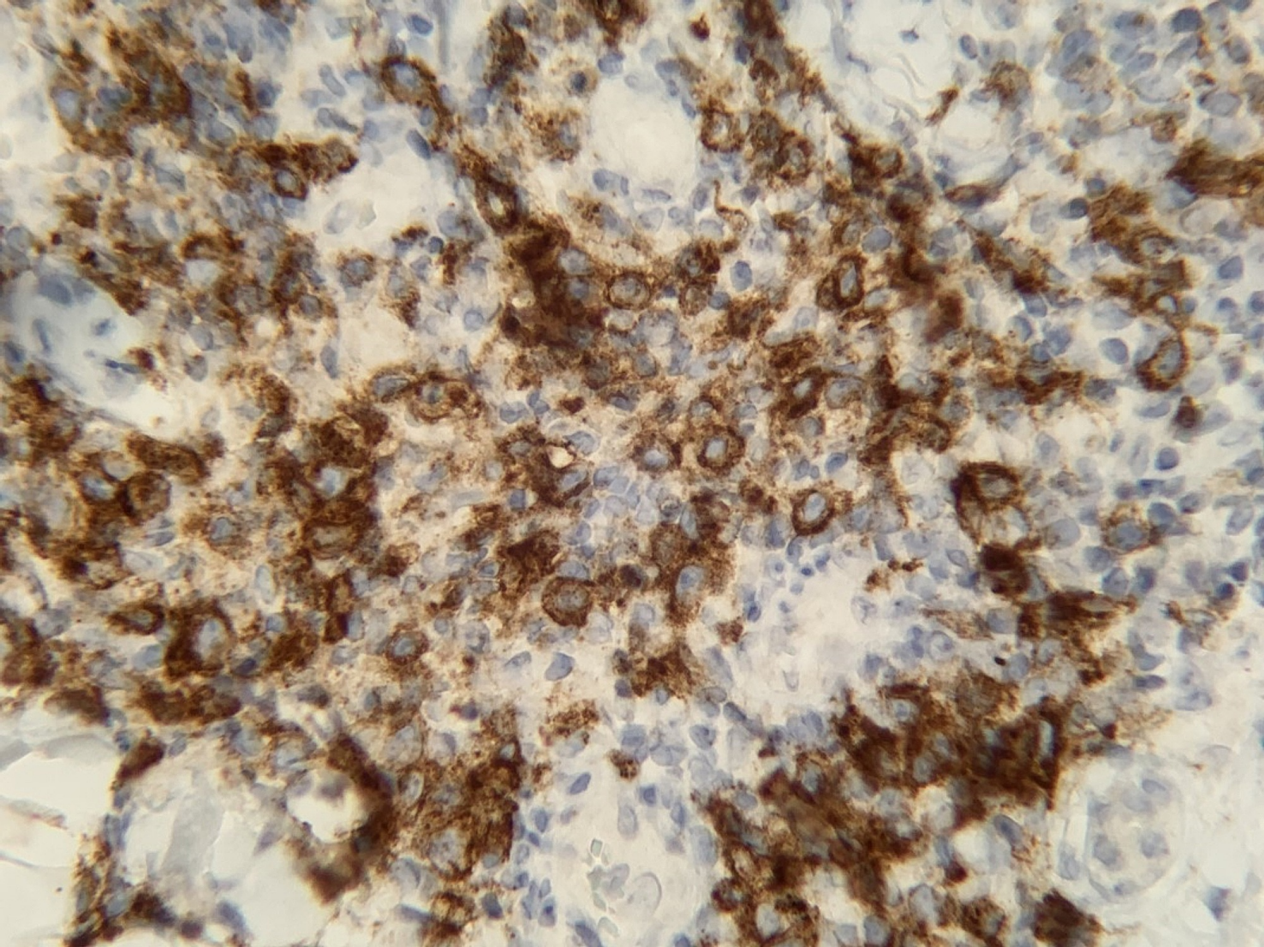
Skin punch biopsy, revealing CD30+ staining appearing in brown.

**Figure 3:**
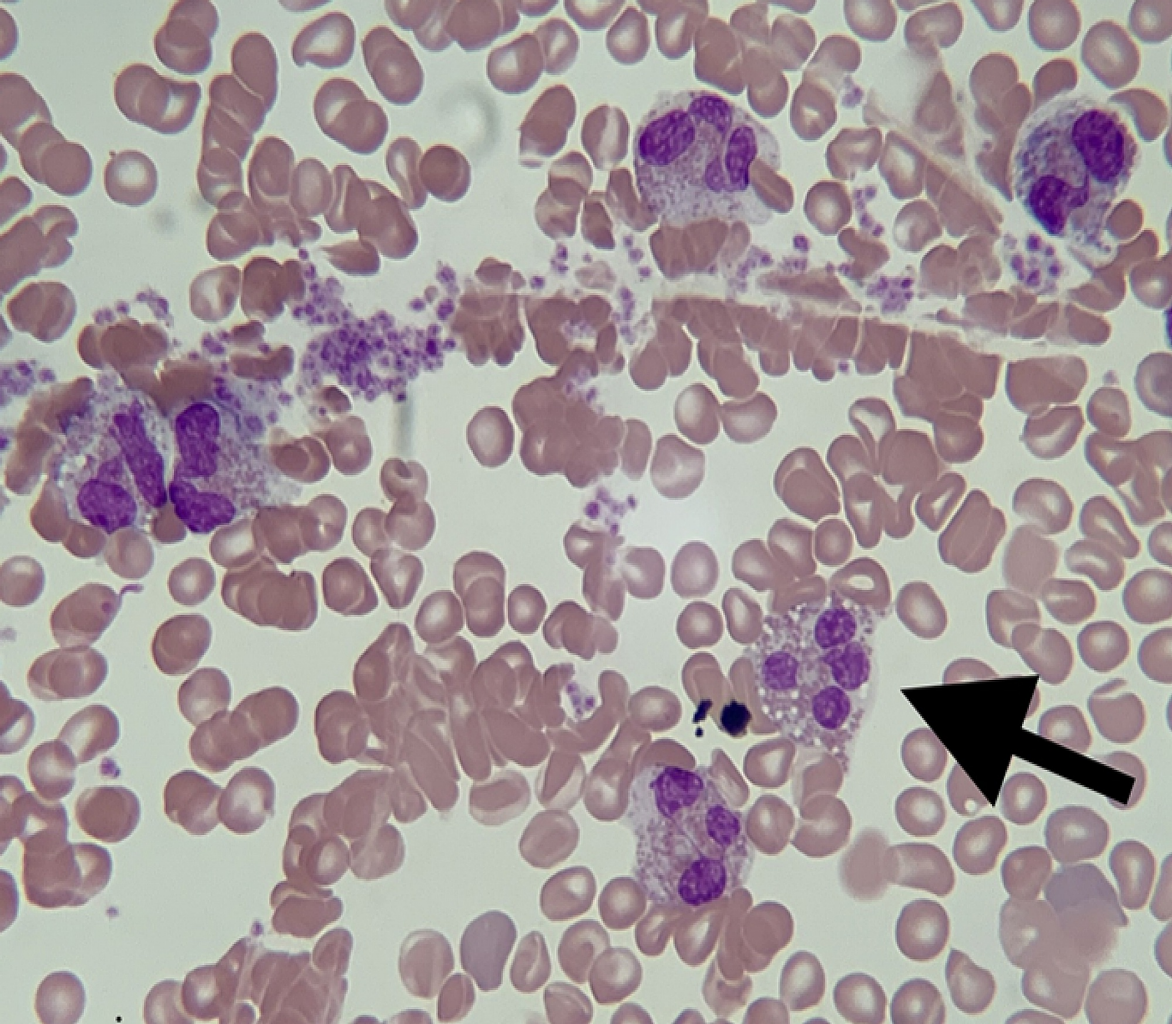
Bone marrow aspirate smear at 1000x magnification revealing atypical eosinophils with hyperlobated nuclei and abnormal granulation (arrow).

During the last hospitalization, the patient’s symptoms were particularly severe, with cough leading to a syncopal episode and hemoptysis with a rise in cardiac biomarkers. He was transferred to our institution for further pulmonary evaluation. Upon arrival, he presented with pleuritic chest pain, dry cough, anorexia, and diffuse pruritic rash. His vital signs were unremarkable except for tachycardia with a heart rate of 103 beats per minute. Physical examination revealed a chronically ill-appearing man in moderate distress. Scattered erythematous papules that were in various stages of healing were noted on his extremities, some having central ulceration and crusting (Figure 1). Notable laboratory investigations included an AEC of 9.20 K/uL (reference range: 0.00-0.70 K/uL) and an elevated tryptase level at 16 ug/L despite ongoing prednisone use. Laboratory tests for infectious and rheumatologic causes were unrevealing. A skin punch biopsy showed a dense perivascular and interstitial lymphoid Infiltrate with high levels of CD30+ cells consistent with lymphomatoid papulosis (Figure 2). Results from bone marrow aspiration and biopsy confirmed chronic eosinophilic leukemia (Figure 3). Fluorescence in situ hybridization (FISH) analysis detected a cryptic deletion on chromosome 4q (CHIC2 gene) indicative of the FIP1L1-PDGFRA gene fusion.

Daily imatinib 400 mg was initiated and prednisone was continued due to concern for cardiac involvement despite normal trans-thoracic echocardiogram. Within one day of imatinib initiation, the patient’s eosinophilia normalized and he was discharged home in stable condition. At the follow-up examination four weeks later, all his symptoms had resolved. He reached both hematologic, cytogenetic, and molecular remission within three months of treatment. Prednisone was tapered off and his imatinib dose was decreased to 100 mg daily.

## Discussion

HE can be primary (clonal), secondary to other etiologies, or idiopathic [3]. Once secondary causes of eosinophilia have been evaluated and excluded, laboratory evaluation of primary eosinophilia should begin. Regardless of etiology, HE can have a myriad of manifestations, including dermatologic, pulmonary, gastrointestinal, cardiac, and neurologic symptoms resulting from eosinophil-derived substances released from eosinophil granules that can cause tissue damage [3,4].

Clonal eosinophilia is most frequently associated with chronic myeloid neoplasms. Chronic eosinophilic leukemia is a rare chronic myeloproliferative disease resulting from clonal proliferation of eosinophil precursors. The 2008 World Health Organization classification of tumors of hematopoietic and lymphoid tissues introduced a new category of myeloid and lymphoid neoplasms characterized by eosinophilia and abnormalities in the PDGFRA, PDGFRB or FGFR1 genes [5]. The FIP1L1-PDGFRA fusion caused by a cryptic deletion of 800 kilobase pairs on chromosome 4q12 yields a constitutively active tyrosine kinase that stimulates eosinophil proliferation, manifesting as chronic eosinophilic leukemia [6].

The presence of dysplastic eosinophils may suggest clonal eosinophilia, and elevated serum tryptase and vitamin B12 levels have been observed with myeloid neoplasms, particularly in those with PDGFRA or PDGFRB fusion genes [7]. Clinical manifestations of chronic eosinophilic leukemia are multisystemic due to tissue infiltration by neoplastic eosinophils that cause variable tissue damage. In particular, restrictive cardiomyopathy secondary to endomyocardial fibrosis has been reported in chronic eosinophilic leukemia and carries a poor prognosis [5].

Dermatologic manifestations from PDGFRA-associated hypereosinophilic syndrome are common and can include dermatitis and urticaria. Lymphomatoid papulosis with erythematous papulonodular lesions is a recurrent skin eruption, which can become necrotic. It has histopathologic features resembling lymphoma [8], and about 10%-20% of cases can be associated with systemic lymphoproliferative disorders. Histopathology of the lesions show large atypical CD30+ lymphocytes with pale eosinophilic cytoplasm, large irregular nuclei, open vesicular chromatin, and a prominent nucleolus [9]. Several case reports have described the association of lymphomatoid papulosis with hypereosinophilia and successful treatment of PDGFRA associated chronic eosinophilic leukemia with imatinib [8,10,11].

Patients with documented rearrangements or mutations involving PDGFRA should be treated with imatinib mesylate (100-400 mg by mouth daily). The response to imatinib is rapid (within days), and complete hematologic and molecular remission is almost universal [2]. In our patient, the delayed diagnosis of chronic eosinophilic leukemia, which is highly treatable, could have led to transformation to a more aggressive disease and irreversible end-organ dysfunction if treatment had been delayed any longer.

## Conclusions

HE can be indicative of chronic eosinophilic leukemia, a rare hematologic malignancy, which can often be mistaken for more common conditions such as asthma, pneumonia, allergies, cardiac disease, and others. While chronic eosinophilic leukemia can be treatable, end-organ failure and severe outcomes will ensue if left untreated. Therefore, physicians should be wary of anchoring bias, where uncommon causes of common presentations are not considered, such as occurred in our patient with HE.
